# Prevalence of Nonadherence to Medications among Geriatric Patients in Al-Madinah Al-Munawara City's Hospitals, Kingdom of Saudi Arabia

**DOI:** 10.1155/2023/3312310

**Published:** 2023-10-31

**Authors:** Haifa Abdulrahman Fadil, Waad Abdulkareem Sammman, Riham Mohamed Elshafie

**Affiliations:** ^1^Department of Clinical and Hospital Pharmacy, Faculty of Pharmacy, Taibah University, Almadinah Almunawarah 30078, Saudi Arabia; ^2^Department of Pharmacology, Faculty of Pharmacy, Taibah University, Almadinah Almunawarah 30078, Saudi Arabia; ^3^Clinical Pharmacy Department, ASUSH, Ain Shams University, Cairo, Egypt

## Abstract

**Introduction:**

Medication adherence is a major component in the treatment of chronic patients, especially the older adults. It acts as an essential part of a successful therapeutic outcome, increasing treatment benefits, and decreasing hospital admissions, health care utilization, and cost. Limited research regarding medication adherence has been conducted nationwide; however, data among the older adults is sparse due to the lack of research on this topic. Therefore, this study aimed to determine the prevalence of medication nonadherence among older adults' Saudi patients with chronic diseases living in Al-Madinah Al-Munawwarah city.

**Method:**

A cross-sectional study was conducted on outpatient older patients with chronic diseases who visited clinics at different hospitals in Al-Madinah Al-Munawwarah city, from March until December 2022. The questionnaire was published in a conversational Arabic dialect. The adherence level was measured by the Adherence to Refills and Medications Scale (ARMS) and the reasons for medication nonadherence were collected by using the Cluig scale.

**Results:**

65.1% of females and 34.9% of males have participated in this study. According to the ARMS scale, 67.9% of patients had poor medication adherence and 32.1% had good medication adherence. Poor adherence was higher in females compared to males, with a *p* value = 0.018. For educational level, the ARMS score in graduates was higher than patients who finished middle school and illiterate patients. Based on the Cluig scale, the most common reasons for medication nonadherence were forgetfulness, feeling well, polypharmacy, and subsequent avoidance of side effects. The findings indicate the gender-based differences in the factors that associated with medication nonadherence among older adults.

**Conclusion:**

Medication adherence is crucial especially among older adults to improve clinical outcomes. The current results highlight the prevalence of medication adherence among the older adult population. This result shows the gender-based differences in the factors associated with medication nonadherence among the older adult population.

## 1. Introduction

Medication nonadherence can have a significant impact on the clinical outcomes, such as disease progression, increased healthcare costs, and even death [1]. The benefits of current medical care are substantially undermined by nonadherence, which is a growing concern owing to the increased number of available medications [2]. Older adult patients who disregard medical advice and treatment are more likely to experience therapeutic failure and unintended consequences, which can increase the healthcare costs, cause impairment, and result in early death [3]. The number of people aged 60 years and older is rapidly increasing worldwide. According to the General Authority for Statistics in Saudi Arabia's most recent survey, older adults currently account for 4.19% of the country's total population, and this proportion is projected to reach 18.4% by 2050 [4, 5].

Around 50% of people with chronic conditions do not follow their drug treatment regimen [6]. In the United States healthcare system, nonadherence to the medication regimen is a serious issue that increases the healthcare costs to more than $170 billion annually [7]. Patient-related issues, such as misconceptions about their medications or negative thoughts and/or perceptions about pharmaceuticals, are among the factors that contribute to the nonadherence to therapeutic recommendations [8]. Comorbidities and cognitive impairment, which are highly common in older adult patients, are the factors that could contribute to medication nonadherence [9]. Additionally, older persons have a high prevalence of polypharmacy and experience complexity of treatment plans, and these factors are more frequently linked to poor treatment adherence [10, 11]. Nonadherence to therapy recommendations may also be influenced by socioeconomic factors, such as older age (>60 years), lack of resources, and low health literacy [10, 11].

The Riyadh-based study investigating the adherence rate to long-term therapies and views about medications reported that 56.9% of the participants had low adherence, which was associated with the patients' beliefs and lack of knowledge about their medications [12, 13]. A Saudi-based cross-sectional, national representative household survey of 10,735 people aged 15 years and older reported that only 32.7% of prescribed medications were taken exactly as directed in 2013 [14]. Approximately 64.9% of the 422 older outpatients with chronic conditions who participated in a recent cross-sectional study conducted at King Saud University Medical City, Riyad, Saudi Arabia, showed good medication adherence [15]. Several cross-sectional studies conducted through social media and in hospitals in different regions of Saudi Arabia have investigated the medication nonadherence rate in adult patients aged ≥18 years with one or more chronic diseases, including diabetes mellitus (DM), hypertension, heart diseases, dyslipidemia, asthma, chronic obstructive pulmonary disease, and epilepsy; results ranged from 22% to 96.62% [13, 16–18].

The overwhelming majority of these previous studies investigating the prevalence of medication nonadherence were conducted in adult Saudi patients with one or more chronic diseases. However, only a few studies have specifically examined the medication nonadherence of older adult Saudi patients with any disease condition. Therefore, this study aimed to determine the prevalence of nonadherence to the medication regimen among older adult Saudi patients with all types of chronic disease conditions living in Al-Madinah Al-Munawwarah city using a self-administrated questionnaire published in a conversational Arabic dialect.

## 2. Materials and Methods

### 2.1. Design and Setting

This descriptive cross-sectional study targeted an outpatient geriatric population comprising individuals with chronic diseases who visited various clinics at six different government hospitals in Al-Madinah Al-Munawwarah city, Kingdom of Saudi Arabia.

### 2.2. Sample Size and Study Population

The sample size was calculated using the online Sample Size Calculator (Raosoft, Inc.) based on a population estimate of 20000 older adults in Madinah city from a published report on the Saudi census. The confidence interval (CI) was set at 95% with a margin of error of 5% [19]. Therefore, a minimum possible effective sample size of 377 was required to achieve the study's objectives [20]. A convenience sampling procedure was used. Participants were considered eligible if they had attended the outpatient clinics at the different hospitals, were more than 60 years old based on the Saudi Census definition, spoke Arabic, and had taken at least one medicine for a chronic illness [19]. The caregivers of the older adults were requested to help complete the survey questionnaire for patients with cognitive impairment, psychiatric diseases, hearing impairment, or communication problems.

Written informed consent was obtained from all the patients, and the study was conducted by the 1964 Declaration of Helsinki and its comparable ethical standards or later amendments. Ethical approval was obtained from the Institutional Review Board of Tiabah University College of Pharmacy (TUCDREC/20190403). All the participants were informed about the study's objective. Only participants who agreed to participate were given a questionnaire. The privacy of the study participants was rigorously protected throughout the data-gathering process, and each participant was assigned an anonymized serial number. Analyses were carried out on anonymized data, with encryption using IBM SPSS 28.

### 2.3. Data Collection

The data were collected from March until December 2022. Data collectors and social media channels were used to distribute the questionnaire. The adherence level was measured by conducting a 5-minute survey using the Adherence to Refills and Medications Scale (ARMS) published in the Arabic language, which includes 12 items. This questionnaire has high performance characteristics even among patients with poor literacy levels and is a valid and reliable medication adherence scale utilized in a chronic disease population [21]. Furthermore, several studies have shown that ARMS (Arabic version) is a valid and reliable tool for measuring adherence to chronic medications among native Arabic speaking patients [22–25]. The scale has demonstrated a high internal consistency reliability, with a Cronbach's alpha value of 0.856. Four items were used to assess the adherence to refilling medications on schedule, while the remaining eight items assess the adherence to taking medications. Each item is scored on a 4-point Likert scale (1 = none, 2 = some, 3 = most, and 4 = all). The total score ranges from 12 to 48, with higher scores indicating poorer adherence. Furthermore, a score of ≥16 was used as a cut-off point to categorize surveyed patients into nonadherent (e.g., ≥16) and adherent (e.g., <16). The reasons for medication nonadherence were determined using the Cluig scale [26, 27]. This final 16-item scale had good internal consistency reliability (Cronbach's alpha = 0.89) [28]. The Arabic version of the instrument showed acceptable reliability [29, 30]. These answers were used to analyze the impact of each reason for nonadherence. To utilize both scales in this study, license agreements were signed and permissions were requested from the appropriate authorities. Numerous faculty members pilot-tested the study questionnaire and reviewed it for comprehensiveness, type, flow, and clarity; no adjustments were deemed necessary. Data were collected and analyzed using IBM SPSS 28 for Windows software, and a *P* value of <0.05 was considered significant.

### 2.4. Statistical Analysis

Categorical variables were expressed as numbers and percentages, while continuous variables were expressed as means and standard deviations.

Independent sample *t*-tests and one-way analysis of variance (ANOVA) were used to evaluate the association between ARMS scores and participant characteristics. To determine the influence of factors related to participant medication adherence, multivariable logistic regression was performed. IBM SPSS 28 for Windows software was used to perform all analyses, and a *P* value of <0.05 was considered significant.

## 3. Results

### 3.1. Characteristics of the Participants

A total of 501 participants participated in this study. Of them, 65.1% were women, while 34.9% were men. Approximately 65.7% of the participants were aged 60–70 years, 24.4% were aged 70–80 years, 8.6% were aged 80–90 years, and 1.4% were aged 90 years and older. Moreover, 36.5% of the participants were illiterate, 25.9% completed a college degree, 22.2% completed high school, and 15.4% completed primary school. Approximately 41% of the patients were taking six or more drugs, 19.5% were taking three drugs, 15.1% were taking four drugs, 11.2% were taking two drugs, and 2.4% were taking one drug. The sociodemographic characteristics of the participants are presented in Table 1.

### 3.2. Adherence Prevalence

According to the ARMS scale, 67.9% of the patients had poor medication adherence, while 32.1% had good medication adherence, as shown in Table 2.

Independent *t*-tests and one-way ANOVA were used to determine the associations between ARMS scores and patient characteristics. Gender and education level showed statistically significant results. The ARMS score was higher in women than in men (*P* = 0.018) as shown in Table 3. With regard to the educational level, the ARMS score of college graduates (mean = 22.88) was higher than that of patients who finished middle school (mean = 19.87) and illiterate patients (mean = 20.57), while no significant difference was found between illiterate and patients who finished primary and middle school (*P* = 0.004) (Table 3).

**Figure 1:**
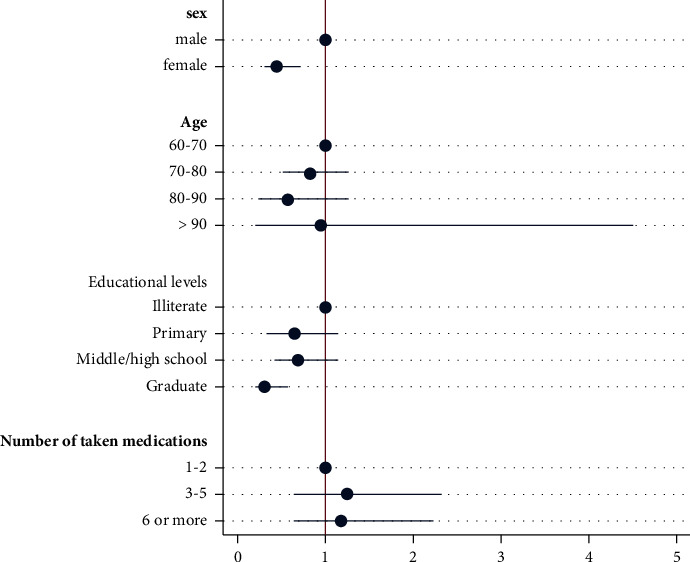
Odds ratio of factors associated with medication adherence.

Simple and multivariable logistic regression analyses were performed to identify the independent predictors of medication adherence among participants. Women were less likely to adhere to the medication regimen than men (odds ratio (OR) = 0.46, 95% confidence interval (CI): 0.30–0.71, *P* < 0.001). In terms of educational level, university graduates were less likely to adhere to the medication regimen than illiterate participants (OR = 0.32, 95% CI: 0.18–0.56, *P* < 0.001) (Table 4, Figure 1).

### 3.3. Reasons for Nonadherence to Medications

**Figure 2:**
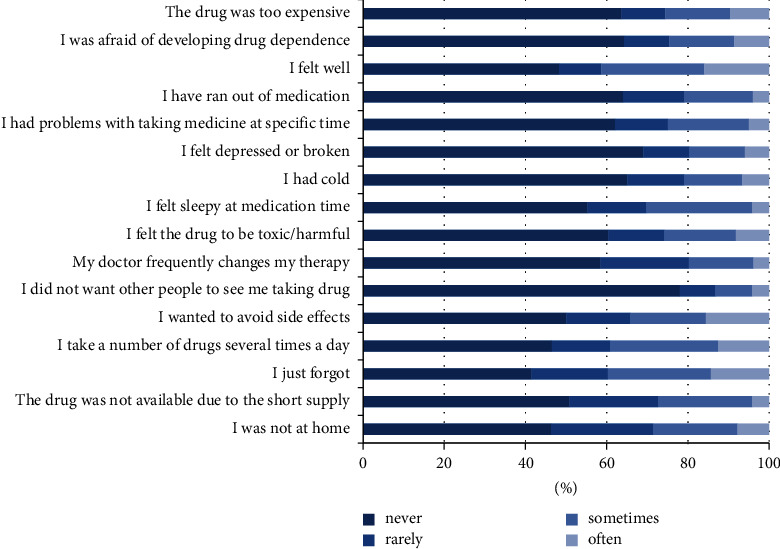
Mean number of participants according to the cause of medication nonadherence.

Culig's medication adherence scale was used to assess medication adherence based on the reasons provided by the participants. The most common reason for nonadherence was forgetfulness (mean = 1.13), followed by feeling well (mean = 1.09) and taking a number of drugs several times a day (mean = 1.05) (Figure 2). The least reported reason was not wanting other people to see them take the drug (mean = 0.39) as represented in Figure 1. Furthermore, the Mann–Whitney test was used to compare the causes of nonadherence between men and women. Women obtained higher scores in the “forgetting to take the medication” domain compared with men (*P* = 0.019). Women also obtained higher scores in the “taking a number of drugs several times a day” domain (median = 1) compared with men (median = 0) (*P* = 0.006). Women obtained higher scores in the “desire to avoid side effects” domain (median = 1) compared with men (median = 0) (*P* = 0.015).

Lastly, women obtained higher scores in the “feeling well” domain (median = 1) compared with men (median = 0) (*P*=0.007; Table 5).

## 4. Discussion

Older adults develop multiple chronic health conditions and comorbidities that lead them to take multiple medications for treatment [31]. The patient's adherence to the medication is important for the effective treatment of chronic disease, and nonadherence to the medication regimen among older adult patients contributes to the deterioration in health, high healthcare costs, and high burden among healthcare professionals [13, 15, 32]. Hence, this study aimed to determine the prevalence of medication nonadherence among older adult Saudi patients with various types of disease conditions who were residing in Madinah city. A previous review study on medication adherence reported that in Middle Eastern countries, medication nonadherence ranged from 1.4% to 88% [33]. The current results revealed that over two-thirds of the study participants (68%) were nonadherent to their medications. Although several previous studies conducted in different cities in Saudi Arabia showed variations in the medication nonadherent prevalence rates, this study finding was comparable with most of the studies that reported a high rate of medication nonadherence [13, 34–36]. Unlike our findings, a recent clinic‐based descriptive study evaluating nonadherence to medications among adult individuals with CVDs in Madinah showed that 26% of the patients were nonadherent to their medications [29]. Alongside Alhabib et al.'s study conducted in Riyadh, 64.9% of the older adult individuals showed a high level of medication adherence, good level of adherence in 21.3% of cases, partial level of adherence in 13.3% of cases, and poor level of adherence in 0.5% of cases [15]. This variation in the level of adherence could be attributed to the fact that this study was conducted in one tertiary cardiac center in Al-Madinah. However, the study sample included participants from different treatment sites.

In the present study, medication nonadherence was correlated with patient's sex. Our findings suggest that a high proportion of male patients had slightly better adherence scores compared with female patients. Studies conducted locally and abroad examining gender variations in medication adherence prevalence have produced conflicting findings [29, 37, 38]. This study, along with a few other studies conducted in Saudi Arabia, revealed high levels of medication nonadherence among female patients [13, 15]. Research on gender variations in medication adherence revealed that women were more likely to have several prescriptions, lack monitoring, and have noncompliance with the recommendations of healthcare providers [39, 40]. These variables may partly influence women's decision to not adhere to their prescribed medications. Another study showed that the medication nonadherence rate among men and women was related to the type of disease. According to these results, women were more likely than men to take their diabetes and cardiovascular medications as prescribed [41]. This difference may be explained by the fact that the physiologic variations between men and women have an impact on drug activity, including pharmacokinetics and pharmacodynamics, thus increasing the prevalence of adverse reactions in women compared with that in men [40, 42].

### 4.1. Age

Although the present study was conducted among older adult individuals who were more than 60 years old in the city, age was not significantly associated with the adherence level [19]. In line with this result, no significant difference was observed between low and high medication adherence among older adults [38, 43, 44]. Contrary to this result, a few studies conducted in Saudi Arabia have reported better adherence rates among older adults compared with that among younger adults [15, 45]. However, advancing aging is related to the level of medication nonadherence. Previous studies indicated that poor cognition, high financial burden due to polypharmacy, differences in prescribing dosages, and worrying about the medication side effects increased the medication adherence level among older adult patients [46].

### 4.2. Education Level

The results also revealed a significant positive correlation between education level and medication nonadherence. Similarly, another study found that patients seemed to adhere to their prescribed diabetes medications less even when they were educated and knowledgeable about their treatment regimen [47]. Additionally, according to certain research, patients with lower education level had higher rates of adherence because they had greater trust in medical recommendations. Moreover, the level of understanding regarding the potential side effects of treatment is one of the factors that influence the medication nonadherence among individuals with a higher education level [48, 49]. Inconsistent with this finding, Al-Asmari et al. revealed that uneducated or less educated participants were more likely to be nonadherent compared with those who had secondary‐level or graduate-level education. Additionally, low educational level is associated with poor adherence to the medication regimen [38, 44, 50, 51]. However, some studies across the Middle East reported no differences in the adherence rates according to the educational status [15, 34, 52, 53].

### 4.3. Number of Medications

In this study, the number of medications taken was not associated with the adherence level. The same result was reported in another study conducted in Saudi Arabia regarding the predictors of medication adherence and blood pressure control [45]. Previous studies reported conflicting findings regarding the relationship between the number of prescriptions and the adherence level. Another Saudi Arabian study found that patients with chronic illnesses, several prescriptions, and complex regimens were more likely to have better medication adherence level [12]. By contrast, numerous studies have demonstrated that polypharmacy significantly contributes to medication nonadherence. By reducing the number of prescribed medications and simplifying the management strategy, patient adherence could be improved [13, 18, 54, 55].

### 4.4. Reasons for Nonadherence

This study additionally explored the reasons for nonadherence among older adult patients in Madinah city. The results highlighted that the most common reason for medication nonadherence was forgetfulness, followed by feeling well, polypharmacy, and subsequent avoidance of side effects. The last reported reason was not wanting other people to see them take the drug. These reasons were comparable with those reported by Al‐Asmari et al. who found that the main reasons for medication nonadherence among cardiovascular disease patients were not being at home at the appropriate time, forgetfulness, subsequent avoidance of side effects, and frequent medication changes [29]. Alsolami et al. conducted a study in Saudi Arabia and found that polypharmacy was the primary reason for medication nonadherence, followed by forgetfulness among patients with chronic disease [13]. Another study discussed the factors affecting the adherence to antihypertensive medications in Saudi Arabia. They reported that the primary reason for nonadherence to the antihypertensive medications was their conviction that taking such medication was harmful, since it had a negative impact on their health [45]. An important finding of the current study is the variations in the reasons for medication nonadherence across sex. Forgetfulness, polypharmacy, fear of adverse effects, and feeling well were more likely to occur among women. This result highlights the gender-based differences in the factors of medication nonadherence in the older adult population.

Adherence to the medications is crucial, especially in the older adult population, to optimize clinical outcomes. The current results shed light on the prevalence of medication adherence among the older adult population. Older women and patients with advanced education levels are at a considerable risk of nonadherence to their medication. The varied reasons for nonadherence provided by the participants of the present study, as well as the findings of previous studies, emphasize the need to improve patient education, especially among those who were at risk of nonadherence to their medications. Hence, healthcare providers, especially pharmacists, should be knowledgeable about these reasons. Furthermore, pharmacists play a significant role in implementing medication adherence interventions that are tailored to the needs of the community. Pharmacists should direct patients' education and intervention to minimize such reasons (e.g., forgetfulness and concerns) and, consequently, reduce the medication nonadherence rate. Accordingly, different strategies should be utilized to enhance the medication adherence among older adult patients: provision of patient education according to the health literacy level, application of medication adherence enhancement interventions such as a pictogram and medication reminders, and simplifying the medication regimen to once daily to accommodate the patient needs. In addition, the results highlight that older adult women are at considerable risk of medication nonadherence. Therefore, female patients should be encouraged to pick up their medication, discuss their concerns, and obtain appropriate education.

This study had some limitations. Self-reporting was applied to gather the data for this study, which raises the possibility of social desirability and recall bias. In addition, this was a cross-sectional study, and causality could not be determined. Despite these limitations, the current study focused on older adult patients in the region, which will expand our understanding in this field, as only a few studies have addressed the medication nonadherence among geriatric patients in Saudi Arabia. Therefore, an appropriate understanding of this issue will provide a platform from which necessary steps can be taken, especially by pharmacists, to create and implement interventions to address these challenges. In addition, medication nonadherence is a multidimensional behavior that is affected by various factors. Further qualitative studies are required to address the complexity of barriers to adherence.

## 5. Conclusion

Medication adherence is essential to optimize clinical outcomes, particularly for older adults. The present findings highlight the prevalence and reasons for medication nonadherence among older adult population. A 68% nonadherence rate was observed among patients, with gender and education level significantly associated with nonadherence. In addition, the main associated factors of nonadherence were forgetfulness, feeling well, and taking multiple medications. The study suggests the need for interventions to improve medication adherence and health outcomes among older Saudi patients, considering their demographic and medication characteristics. Longitudinal studies are recommended for future validation.

## Figures and Tables

**Table 1:** Characteristics of the participants (*N* = 501).

	*N*	%

*Gender*		
Male	175	34.9
Female	326	65.1

*Age*		
60–70 years	329	65.7
70–80 years	122	24.4
80–90 years	43	8.6
≥90 years	7	1.4

*Education level*		
Illiterate	183	36.5
Primary	77	15.4
Middle/high school	111	22.2
College graduate	130	25.9

*Number of medications taken*		
1	12	2.4
2	56	11.2
3	98	19.5
4	76	15.1
5	54	10.8
6 or more	206	41.0

**Table 2:** Medication adherence among patients (*N* = 501).

	*N*	%	95% CI	
			Lower	Upper
Nonadherent	340	67.9	63.7	72.1
Adherent	161	32.1	27.9	36.3

CI: confidence interval.

**Table 3:** Association between ARMS score and characteristics of patients.

	Mean	SD	*P* value

*Gender*			**0.018**
Male	20.09	6.60	
Female	21.62	6.99	

*Age*			0.422
60–70 years	21.11	6.79	
70–80 years	20.80	7.12	
80–90 years	21.02	6.37	
>90	25.29	10.06	

*Education level*			**0.004**
Illiterate	20.57	7.56	
Primary	21.03	6.65	
Middle/high school	19.87	5.99	
Graduate	22.88	6.45	

*Number of taken medication*			0.746
1-2	21.66	7.44	
3–5	20.93	6.56	
6 or more	21.06	7.07	

ARMS: adherence to refills and medications scale. Bold values are considered significant as they are <0.05.

**Table 4:** Logistic regression of the factors associated with medication adherence.

	Bivariable variate				Multivariable			
	OR	*P* value	95% CI for OR		OR	*P* value	95% CI for OR	
			Lower	Upper			Lower	Upper
*Gender*								
Male	1				1.00			
Female	0.58	**0.006**	0.39	0.86	0.46	**<0.001**	0.30	0.71

*Educational level*								
Illiterate	1.00				1.00			
Primary school	0.73	0.279	0.41	1.29	0.65	0.143	0.36	1.16
Middle\high school	0.91	0.704	0.56	1.48	0.69	0.174	0.41	1.18
Graduate	0.42	**0.001**	0.25	0.71	0.32	**<0.001**	0.18	0.56

*Age*								
60–70 years	1.00				1.00			
70–80 years	1.03	0.909	0.66	1.60	0.82	0.409	0.51	1.31
80–90 years	0.81	0.568	0.40	1.65	0.58	0.159	0.27	1.24
≥90 years	1.58	0.555	0.35	7.18	0.95	0.946	0.20	4.53

*Number of taken medication*								
1-2	1.00				1.00			
3–5	1.39	0.287	0.76	2.54	1.25	0.482	0.67	2.33
6 or more	1.35	0.339	0.73	2.49	1.19	0.595	0.63	2.24

CI: confidence interval; OR: odds ratio. Bold values are considered significant as they are <0.05.

**Table 5:** Causes of nonadherence across gender.

	Male		Female		*P* value
	Median (IQR)	Mean rank	Median (IQR)	Mean rank	

I was not at home	1 (2)	261.10	1 (2)	245.58	0.222
The drug was not available due to shortage of supply	0 (2)	241.43	1 (2)	256.14	0.238
I just forgot	1 (2)	231.35	1 (2)	261.55	**0.019**
I take a number of drugs several times a day	0 (2)	228.47	1 (2)	263.09	**0.006**
I wanted to avoid the side effects	0 (2)	231.09	1 (2)	261.69	**0.015**
I did not want other people to see me taking a medication	0 (0)	250.14	0 (0)	251.46	0.893
My doctor frequently changes my treatment	0 (1)	252.66	0 (1)	250.11	0.832
I felt that the drug is toxic/harmful	0 (1)	250.40	0 (2)	251.32	0.938
I felt sleepy during medication time	0 (2)	248.17	0 (2)	252.52	0.722
I had colds	0 (1)	245.41	0 (1)	254.00	0.455
I felt depressed or broken	0 (1)	244.54	0 (1)	254.47	0.370
I had problems with taking a medication at a specific time	0 (1)	252.42	0 (2)	250.24	0.853
I ran out of medications	0 (1)	248.71	0 (0)	252.23	0.761
I felt well	0 (2)	228.77	1 (2)	262.93	**0.007**
I was afraid of developing drug dependence	0 (1)	242.63	0 (2)	255.49	0.267
The drug was too expensive	0 (1)	247.69	0 (2)	252.78	0.662

IQR: interquartile range. Bold values are considered significant as they are <0.05.

## Data Availability

Data used in this study are available from the corresponding author upon request.
